# Partial Androgen Insensitivity Syndrome and Congenital Adrenal Hyperplasia—A Case Report of the Coexistence of Two Rare Diseases in One Patient

**DOI:** 10.3390/reports8040212

**Published:** 2025-10-23

**Authors:** Mariola Krzyścin, Agnieszka Brodowska, Gabriela Furtak, Dominika Pietrzyk, Katarzyna Zając, Bartosz Oder, Adam Przepiera, Elżbieta Sowińska-Przepiera

**Affiliations:** 1 Pediatric, Adolescent Gynecology Clinic, Department of Gynecology, Endocrinology and Gynecological Oncology, Pomeranian Medical University in Szczecin, Unii Lubelskiej 1, 71-252 Szczecin, Poland; drkrzyscin@gmail.com (M.K.); dominikapietrzyk.lote@gmail.com (D.P.); bartek.oder@gmail.com (B.O.); 2 Department of Gynecology, Endocrinology and Gynecological Oncology, Pomeranian Medical University in Szczecin, Unii Lubelskiej 1, 71-252 Szczecin, Poland; agnieszka.brodowska@pum.edu.pl; 3 Department of Reconstructive Surgery and Gynecological Oncology, Pomeranian Medical University in Szczecin, Powstańców Wielkopolskich 72, 70-111 Szczecin, Poland; gabrysiafurtak@wp.pl; 4 Department of Urology and Urologic Oncology, Pomeranian Medical University in Szczecin, Powstańców Wielkopolskich 72, 70-111 Szczecin, Poland; adam.przepiera@pum.edu.pl; 5 Individual Laboratory for Endocrine Diagnostics, Pomeranian Medical University in Szczecin, Unii Lubelskiej 1, 71-252 Szczecin, Poland

**Keywords:** partial androgen insensitivity syndrome, congenital adrenal hyperplasia, disorders of sex development, P450 oxidoreductase deficiency, gonadectomy

## Abstract

**Background and Clinical Significance**: In a single phenotypically female patient, we describe the rare co-occurrence of partial androgen insensitivity syndrome (PAIS) and congenital adrenal hyperplasia (CAH). Partial androgen insensitivity syndrome (PAIS) is one of disorder of sex development (DSD) with a 46 XY karyotype. Congenital adrenal hyperplasia (CAH) is a genetic defect in adrenal steroidogenesis. **Case presentation**: We present the case of a 26-year-old female patient who was observed to have abnormally formed external genitourinary organs. She was diagnosed at the neonatal period. Tests performed showed a 46 XY karyotype, an absence of sex chromatin with a weakly positive DNA test for the SRY gene, an absence of uterine primordium with the presence of male gonads in the perineal skin folds, and a urethral outlet at the base of an undeveloped genital process. The daily urinary steroid excretion profile was normal. The patient was diagnosed with partial androgen insensitivity syndrome (PAIS). As a 4-year-old child, she underwent a bilateral gonadectomy due to possible further virilization and also the risk of testicular malignancy. Despite treatment, progressive androgenization was observed, the cause of which turned out to be congenital adrenal hyperplasia (CAH) in the course of P450 oxidoreductase (POR) disorder. **Conclusions**: In this article, we highlight the exceptional rarity of the co-occurrence of PAIS and CAH, underscoring the need for a multidisciplinary and individualized approach in the absence of clear guidelines regarding surgical timing and gender identity. Careful clinical evaluation and ongoing observation are essential for accurate diagnosis and optimal patient care.

## 1. Introduction and Clinical Significance

One of disorders of sex development (DSD) with a 46 XY karyotype is androgen insensitivity syndrome (AIS). It is inherited in a recessive manner, coupled to the X chromosome, and determined by a mutation within the gene encoding the androgen receptor. The frequency of AIS is estimated to be about 4.1 per 100,000 live-born children [1]. The manifestation of AIS (androgen insensivity syndrom) is varied, ranging from the mild phenotypically male form of MAIS (mild androgen insensitivity syndrome), to the phenotypic intersex forms of PAIS (partial androgen insensitivity syndrome), to the phenotypically female form of CAIS (complete androgen insensitivity syndrome) with normally differentiated female genitalia.

Partial androgen insensitivity syndrome (PAIS) results from mutations in the androgen receptor gene and is characterized by a wide range of phenotypes. Mutations in the androgen receptor (AR) gene in coding regions have been identified in less than 25% of patients with a clinical diagnosis of PAIS, and such AR mutations remain undetected in a proportion of patients with impaired endogenous AR activity [2]. Six different mutations responsible for partial androgen insensitivity syndrome have been described, including the following: Met742Ile, Met708Ile, Gln798Glu, Arg840Cys, Arg855His, and Ile869Met [3]. Various types of under-masculinization of external genitalia, at birth and during puberty, result from these mutations in the androgen receptor (AR) gene, which is found on the X chromosome [4,5]. The clinical presentation of PAIS depends not only on the level of androgens, but especially on the response of the body to their presence. The typical picture of the external genitalia includes the following: severe hypospadias (scrotal–ovarian), a bipartite scrotum that may contain gonads, micropenis, and clitoral hypertrophy [6].

Congenital adrenal hyperplasia (CSF, CAH) is a genetically determined disorder in which the enzymes/proteins involved in the synthesis of adrenal cortical hormones are disrupted. The essence of CAH is the reduced or absent cortisol available for the glucocorticosteroid receptor. This results in excessive ACTH production and secondary adrenal hypertrophy. Depending on which gene is involved, different forms of CAH also have other abnormalities of adrenal steroidogenesis, resulting directly from reduced enzyme activity and indirectly from the ACTH excess-induced increased synthesis of those compounds in which the enzyme is not involved [7].

We present a case report of a rare co-occurrence of PAIS and CAH in one female patient. Now 26 years old, she was diagnosed in infancy due to ambiguous urogenital organs, the presence of testes, and the absence of a uterus. In this paper, we aim to highlight the rarity and uniqueness of these two diseases’ occurrence at the same time. We emphasize the importance of teamwork, multidisciplinary watchfulness, and comprehensive treatment for the patient, who requires special attention due to ambiguous guidelines regarding the precise moment of surgery and challenges with gender identity.

## 2. Case Presentation

We present a case report of a now 26-year-old female patient, who was diagnosed in early infancy due to the ambiguous presentation of genitourinary organs, as a DSD patient. The following case description is structured chronologically, covering the neonatal period, infancy, childhood, adolescence, and adulthood.

### 2.1. Neonatal Period

**Figure 1:**
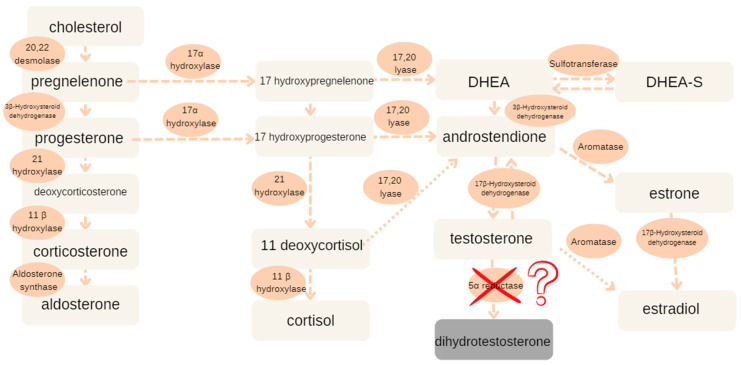
Steroidogenic pathways in our patient, as inferred from the 24 h urinary steroid profile alongside additional biochemical and hormonal test results.

The newborn was delivered at 39 weeks and 1 day of gestation, from the first pregnancy and first delivery, with a birth weight of 2970 g. Apgar scores were five at 1 min, five at 3 min, seven at 5 min, and eight at 10 min. Upon physical examination, she was observed to have a slightly enlarged clitoris, a common urogenital sinus, a blindly terminated vaginal recess, a urethral orifice at the base of an undeveloped genital process, and labioscrotal folds with the bilaterally palpable formations that could constitute gonads on the edge of these folds and the entrance of the inguinal canal. Discrete characteristic traits of dysmorphia such as an asymmetric face, ears anomalies, and fore-ear area were also described. A number of imaging tests were performed and the material for the genetic testing was taken. This showed the presence of the Y body (in the cytogenetic test) and of the SRY gene (in the molecular PCR examination). Additionally, this showed that there was no sex chromatin (in the lining of the mouth cavity), no uterine anlage, and testes were present in the perineum’s skin folds. The interview data on the child’s heritage did not turn up any pathologies that were important for the diagnosis. In hormone tests that were performed on day 14 of life, testosterone and 17-OHP were normal levels for female newborns. A urinary steroid metabolite profile was obtained from a 24 h urine collection performed on the next day (on the 15th day of life of the neonate)**.** The analysis revealed a significant absence of testosterone metabolites, which are typically produced through the pathway involving 5α-reductase. This absence suggests a potential deficiency in 5α-reductase activity, an enzyme critical for the conversion of testosterone to dihydrotestosterone (DHT). As depicted in Figure 1, the results reveal normal concentrations of fetal adrenal steroids, while testosterone metabolites are notably absent, suggesting a potential deficiency in 5α-reductase activity.

**Figure 2:**
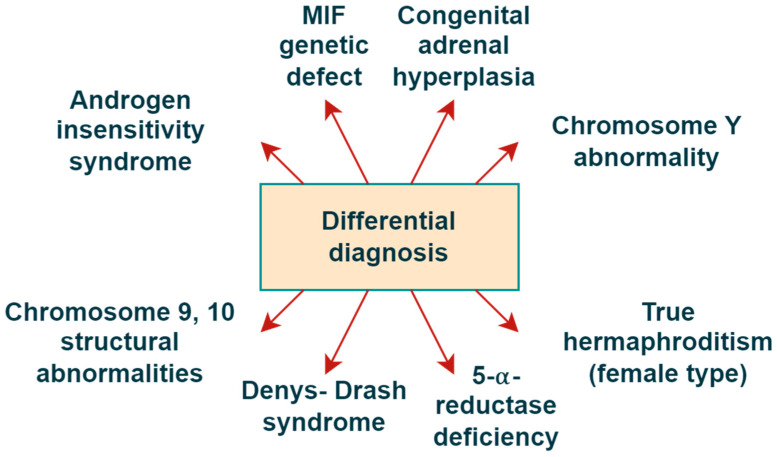
Comprehensive differential diagnosis flowchart.

The schematic illustrates key enzymatic steps involved in steroid biosynthesis, highlighting specific abnormalities identified through diagnostic testing. At this stage, a comprehensive differential diagnosis was considered to encompass conditions such as a Müllerian Inhibiting Factor (MIF) genetic defect, 5α-reductase deficiency, Denys–Drash syndrome, and others depicted in the schematic (Figure 2).

Given the complexity and overlap of symptoms among these disorders, it was deemed prudent to withhold a definitive decision on gender assignment until all diagnostic evaluations, including chromosomal analysis, hormonal assays, and imaging studies, were completed. This approach aligns with current clinical guidelines, which recommend delaying irreversible gender assignment procedures until a thorough assessment is conducted, ensuring that the chosen path aligns with the patient’s best long-term physical and psychological well-being.

### 2.2. Infant

At the age of 2 months, the child was confirmed to have an XY karyotype, and suspicion of androgen insensitivity syndrome (AIS) was raised. After the 46 XY karyotype confirmation, a test of (β-hCG) (Table 1) and an LH-RH test were performed (when the child was 2 months old). The β-hCG stimulation test was performed using Biogonadyl (human chorionic gonadotropin). The patient received intramuscular injections of 500 IU β-hCG once daily for three consecutive days. Serum samples were collected on days 1, 3, and 5 to assess testosterone response, along with additional measurements of estradiol, SHBG, DHEA-SO_4_, and androstenedione. In the test with β-hCG, a slight increase in testosterone and a normal steroid profile were found.

The LH-RH stimulation test was performed by administering a single intravenous dose of gonadotropin-releasing hormone. Blood samples were collected at baseline and at 30, 60, and 90 min post-administration to measure serum levels of luteinizing hormone (LH) and follicle-stimulating hormone (FSH). In the LH-RH test, normal stimulation of LH and FSH secretion was observed. Normal pituitary reserve was also demonstrated. Thus, hypogonadotropic hypogonadism was precluded.

At this stage, it was recommended to refrain from further interventions (sex reassignment surgery) and to observe the patient. Molecular testing of the androgen receptor (AR) gene in the form of an analysis SSCP (single strand confirmation polymorphism) was performed with the approval of the local ethics committee for scientific research after obtaining informed consent from the parents.

### 2.3. Childhood

**Figure 3:**
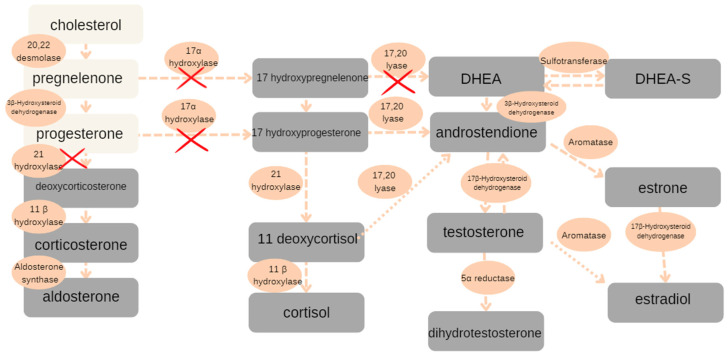
Steroidogenic pathways inferred from the 24 h urinary steroid metabolite profile.

The diagnosis was confirmed by the results of molecular testing when the child’s age was 2 years. Diagnosis was made—androgen insensitivity syndrome (PAIS)—and another genetic study was commissioned. The testing revealed the presence of a mutation in the androgen receptor gene C2754G. The abnormal allele was identified also on the maternal X chromosome. The patient’s mother was found to be a heterozygous carrier, possessing both a normal allele and a mutant allele containing the same exon variant detected in the child. The father was confirmed to have two normal alleles. Due to a marked tendency toward the masculinization of the external genitalia, bilateral gonadectomy was recommended. The increased risk of neoplasm in the dysgenetic gonads with her XY karyotype was also taken into account in the decision-making process. The operation was performed when the child was 4 years old. Macroscopic examination of the postoperative material revealed that Gonad A was oval in shape, measuring 20 × 13 × 10 mm, with an attached spermatic cord measuring 5 cm in length. Gonad B measured 19 × 13 × 10 mm, with a spermatic cord length of 2 cm. Histopathological analysis of the specimens demonstrated that both gonads consisted of immature testicular tissue without any evidence of neoplastic proliferation. Further endocrinological follow-up was recommended. When the patient was 8 years old, a stimulation test with GnRH was performed. Upon physical examination, the patient was assessed as Tanner stage I (M1P1A1), indicating prepubertal development. The GnRH stimulation test was conducted by administering a single intravenous dose of gonadotropin-releasing hormone. Blood samples were collected at baseline and at 30, 60, and 120 min post-administration to measure serum levels of the luteinizing hormone (LH), follicle-stimulating hormone (FSH), estradiol, testosterone, and DHEA-SO_4_. This protocol allowed for the assessment of the hypothalamic–pituitary–gonadal axis function. The values of tropic hormones and sex hormones confirmed the hypergonadotropic hypogonadism caused by the gonadectomy performed. Of the abnormalities in the tests performed, there was an elevated 17-hydroxyprogesterone value with a low androstenedione level. When the patient was 9 years old, due to persistent features of excessive androgenization, despite bilateral gonadectomy and delayed bone age (bone age assessment of the left hand and forearm corresponded to that of a 5-year-9-month-old female), urine steroid metabolite analysis was performed and revealed an abnormal steroid profile, including the presence of delta^5^-pregnenedione, 11-ketopregnanediol, 11-hydroxy-pregnanedione, and atypical pregnenolone metabolites. These findings raised suspicion of congenital adrenal hyperplasia (CAH). Then, in the following week, a 24 h urinary steroid secretion profile was also performed, in which slightly elevated concentrations of 17-OHP, 21-deoxycortisol, progesterone, and corticosterone was observed, with normal androgen and cortisol concentrations. These results indicated a deficiency of P450 oxidoreductase, resulting in partial impairment of both 21-hydroxylase and 17α-hydroxylase activity. Figure 3 presents a schematic representation of the steroid biosynthesis pathway, with the presumed enzymatic deficiencies clearly highlighted.

Molecular testing of the POR gene confirmed cytochrome P450 oxidoreductase deficiency. The presence of the following two mutations was confirmed: frameshift type in exon 13 and missense type in exon 14, and, consequently, this confirmed congenital adrenal hyperplasia.

### 2.4. Adolescence

At the age of 11, estrogen substitution was begun, observing a gradual progression of pubertal development. The patient received psychological care regularly. At the age of 16, separate urethral and vaginal entrances were created, and vulvoplasty was performed. Nearly two years later, vaginal reconstruction was performed with mucosal flaps taken from the oral vestibule, with the recommendation of further vaginal calibration.

### 2.5. Adulthood

**Figure 4:**
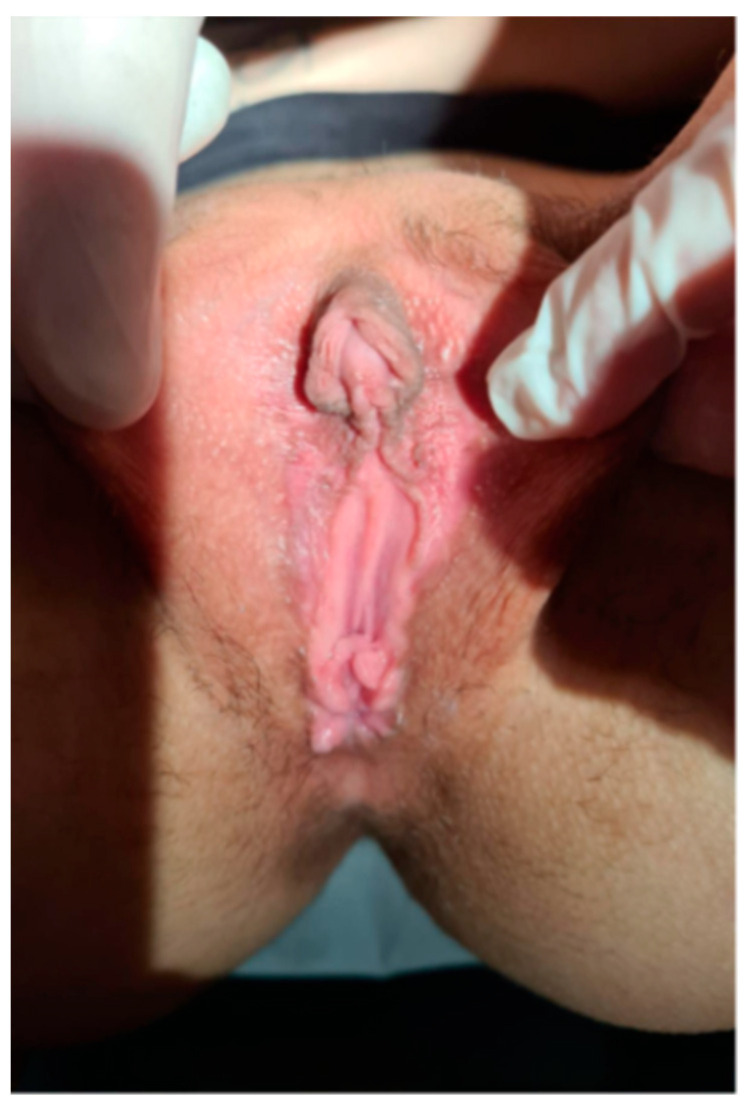
Appearance of external genitalia of the patient with features of partial virilization, including a small phallic structure, partially fused labioscrotal folds, and an external urethral meatus located in the perineal region, below the opening of the surgically created vagina.

Today the patient is 26 years old and is planning a clitoral plastic surgery. When asked about her gender identity, the patient described experiencing gender fluidity, stating that she identifies as female on some days and male on others. She is currently in a stable relationship with a female partner. A current image of the external genitalia (Figure 4) is included in this report, with fully informed consent obtained from the patient, who has reached adulthood.

## 3. Discussion

The androgen insensitivity syndromes (AIS) are phenotypes associated with resistance to the hormonal effects of androgens, and they are classified under the broad classification of 46, XY DSD (disease of sex development). To distinguish between the unique genetic abnormalities that result in the masculinization of the external genitalia, it is necessary to confirm the Y chromosome with karyotype (test for the presence and function of the SRY gene) and conduct research into potential deficiencies in steroidogenesis [8]. A range of androgen activity abnormalities are represented by AIS, which can be further classified into the following three major phenotypes: complete androgen insensitivity syndrome (CAIS), partial androgen insensitivity syndrome (PAIS), and mild androgen insensitivity syndrome (MAIS). The clinical manifestation of AIS varies depending on the AR sensitivity to testosterone and dihydrotestosterone activation, and can be precisely distinguished by the Quigley scale that classifies AIS phenotypes in 7 grades, with 1 grade being described as MAIS and 7 as CAIS. According to Quigley, our patient could be diagnosed as 4 or 5 grade as she revealed characteristic traits for both grades. According to the available literature, most infants diagnosed with PAIS have been assigned the male gender. The surgical correction, of bringing the testicles into the scrotum and hypospadioplasty, is usually performed between 2 and 3 years of age. Not many studies are available on the course of puberty in these patients. Here, we describe a patient with 46, XY DSD who also has linked disease-causing mutations in the POR and AR genes, which are both well-established reasons for undervirilization.

Congenital adrenal hyperplasia (CAH) caused by a mutation in the P450 oxidoreductase (POR) gene is a rare, autosomal recessive disorder. Because cytochrome P450 oxidoreductase deficiency (PORD) affects the synthesis of sex steroids and glucocorticoids, it is a condition of steroid hormone biosynthesis that is closely linked to disorders of sex development (DSD). It can result in infertility, hormonal abnormalities, and ambiguous genitalia in both males and females [9]. The degree of PORD determines the severity of DSD. The association between genotype and phenotype in androgen insensitivity is not clear, and it is well known that different patients may possess varied stages of under-masculinization as a result of the same mutation in the AR gene. In addition to the co-occurrence of two rare diseases, the case of the 26-year-old female patient that we presented is important because PORD is different from other congenital adrenal hyperplasias since it affects numerous enzymes, resulting in their partial activity and a variable hormonal profile. This proves the uniqueness and specificity of the case history we present. Giwercman et al., in their publication, showed the case of a family with two girls who showed mild virilization in relation to their CYP21 genotype. This publication also demonstrated partial insensitivity to androgens, including congenital adrenal hyperplasia and virilization, but there were differences in the location of the disturbances in enzyme activity at various stages of synthesis [10].

In patients with cytochrome P450 oxidoreductase deficiency (PORD), the disorder is due to the decreased activity of several enzymes, including the following: 21-hydroxylase, 17a-hydroxylase, and 17,20-lyase [11]. About 30–40% of patients are still unable to reach a conclusive diagnosis even after thorough diagnostic testing. The course can range from nearly symptomless forms to severe, life-threatening ones, depending on the level of clinical presentation and the levels of enzyme deficiencies [12,13,14]. In our patient, only some discrete characteristic traits of dysmorphia were described, which were as follows: asymmetric face, ears anomalies, and fore-ear area. She revealed some features of the disease characteristic for males and some for females with P450 oxidoreductase deficiency. For males with the condition P450 oxidoreductase deficiency, the characteristic features are a small penis, undescended testicles, and feminization features of the external genitalia, while females are often born with an overgrown vagina, fused labia minora, underdevelopment of the labia majora, clitoromegaly, and primary amenorrhea.

Of the abnormalities in the hormonal tests, a cortisol deficiency, characteristic of CAH, is observed, with normal or slightly elevated ACTH levels [14]. In the CRH test, an excessive increase in ACTH concentration is observed, in comparison to baseline concentration. Typically, elevated concentrations of pregnenolone, progesterone, and their metabolites are also observed, with a characteristic increase in concentration after ACTH stimulation [15,16].

Concentrations of other androgens are usually low and may remain stable or increase slightly under ACTH and chorionic β-gonadotropin stimulation [14,16]. In a daily urine collection of a CAH patient, an increase in specific steroid metabolites (pregnenediol, pregnenadiol, pregnanetriol, pregnanetriolone, 5a-tetrahydrocorticosterone, 11-dehydrometabolites of steroids, and 17a-hydroxypregnanolone) is noted [13]. The mainstay of treatment is glucocorticosteroid substitution, in doses that will restore the normal diurnal rhythm of cortisol or protect the patient in stressful situations. The diagnosis, treatment, and, most importantly, the timing and appropriate assignment of sex, are still very challenging in PAIS patients. So far, no clear-cut management has been established. The literature describes cases of treatment with a short cycle of testosterone (25 mg intramuscularly, every 3 months) or topical treatment with dihydrotestosterone-containing gel, which so far seemed to be helpful in definitive sex assignment [17].

PAIS and CAH are specific genetic disorders that cause abnormal sexual development [18,19]. The majority of cases documented so far have not focused on patients’ fertility, so it is still unclear if POR is linked to human infertility. This is made obvious by the unknown occurrence of POR gene variations and the inadequate quality of fertility-investigating studies [20]. Among the long-term complications of CAH, infertility is frequent in both female and male patients [21].

In order to prevent the effects of future virilization and to lower the risk of testicular cancer in children with undescended testes, the therapeutic regimen for PAIS patients assigned and raised as females suggests bilateral orchiectomy during childhood. These tumors may be germ cell tumors and gonadoblastomas. They may develop into malignancy and often affect 1.5 to 2 percent of undescended testes. In most cases, additional surgery is required, such as genital reconstruction. However, vaginal dilators are an effective first-line treatment for the intensification of the length of a short vagina [22].

In order to promote puberty and guarantee the appropriate development of secondary female sexual characteristics, hormonal treatment using estrogen is required [8].

In patients with DSD, a problem of discrepancy between physical sex and a mental gender–sex that they identify with can be developed [23]. This conflict, leading to gender dysphoria, is nowadays called gender identity disorder (GID) [24]. Adults and teenagers with GID may exhibit the desire to live or be treated as the other sex. Their convictions about their identity, involving a strong feeling of dissatisfaction about oneself as male or female, are strong and persistent [25]. The gender assignment to a DSD infant is still a very challenging task and should involve a multidisciplinary team of specialists and the family [26].

Another scientific systematic review and meta-analysis stated that overall GID prevalence in PAIS adolescents and adults was 15% (21/141), which is equal to the prevalence of GID among all that were under examination in this study—from all DSD groups. There were cases of GID in 12% of PAIS patients raised as females and in 25% of those who were raised as males, but this difference was not statistically significant [24].

One study, with the objective of assessing the quality of life and psychosocial well-being of women with DSD, states that an impaired quality of life and more affective distress were observed, and this was particularly visible in cases of patients with CAH, virilized 46, XX and 46, XY females. Among the possible causes, trauma from distressing diagnostic procedures was enumerated, as well as the chronic illnesses in themselves, or the psychosocial consequences of the disorders [27,28]. Out of concern for DSD patients, it highly crucial to remember that each case is unique, and each patient should be provided with the best multidisciplinary care and long-held psychosexual support to ease their development [24,29,30].

Performing an elective genital operation in DSD pediatric patients is one of many controversial matters regarding DSD patients’ management. Surgical operations for people with PAIS and CAH aim to reduce the risk of late virilization and avoiding the risk of cancer. One of the key issues is the testes, with consideration being given to leaving them until the age of gender determination or removing them to avoid virilization during puberty in individuals who identify as female [31,32].

In order to promote puberty and the development of secondary female sexual characteristics, hormonal treatment with estrogen is needed [8,33]. A consequence of gonadectomy is the development of hypergonadotropic hypogonadism. Therefore, sex steroid replacement is an important component of management for patients with some types of XY DSD, including PAIS. The goals of replacement include the induction of secondary sex characteristics, achievement of optimum bone mineral density, and the optimization and promotion of uterine development [34,35,36]. Hormone replacement can also impact psychosocial and psychosexual development and general well-being [37,38].

## 4. Conclusions

In our article, we would like to draw attention to the rarity of the simultaneous occurrence of these two diseases and emphasize the importance of a multidisciplinary team and a holistic approach to the patient, who requires special care due to the lack of clear guidelines on the timing of surgery and gender identity issues. Infants with PAIS, in contrast to CAIS, typically have ambiguous genitalia at birth, necessitating a thorough diagnosis and a decision regarding sex assignment. Improving the care plan till adulthood requires educating the families of newborns with androgen insensitivity syndrome. More research is required to examine the connection between fertility and the POR genotype. Decisions about gender assignment, fertility, gonadectomy timing, psychological impacts, and genetic counseling are all complicated aspects of diagnosing and treating AIS.

## Figures and Tables

**Table 1:** Results of β-hCG stimulation test (with Biogonadil). β-hCG: human chorionic gonadotropin.

Time (Days)	Testosterone (pg/mL)	Estradiol (pg/mL)	SHBG (nmol/L)	DHEA-SO4 (ng/mL)	Androstendione (ng/dL)
1	<50	<5.5	141	92	12
3	621	<5.5	x	x	x
5	532	<5.5	139	121	10

## Data Availability

The original data presented in the study are included in the article, further inquiries can be directed to the corresponding author.
